# From variant detection to interpretation in idiopathic erythrocytosis: A structured approach applied to a clinical cohort

**DOI:** 10.1111/bjh.70597

**Published:** 2026-06-12

**Authors:** Alessandra Giannella, Fabrizio Vianello, Simone Zoletto, Arianna Magrini, Ilaria Gianesello, Gianni Binotto, Nicolo’ Danesin, Jessica Ceccato, Giulia Gualtiero, Maria Piazza, Samuela Carraro, Valeria Carabotta, Francesco Cinetto, Alessandro Cellini, Marco Carraro, Carmela Gurrieri, Laura Pavan, Francesco Piazza, Andrea Visentin, Walter Ageno, Livio Trentin, Giulio Ceolotto

**Affiliations:** ^1^ Department of Medicine DIMED University of Padua School of Medicine Padua Italy; ^2^ Hematology Unit, Department of Medicine DIMED University of Padua School of Medicine Padua Italy; ^3^ Rare Diseases Referral Center Internal Medicine 1, Ca' Foncello Hospital, AULSS2 Marca Trevigiana Treviso Italy

**Keywords:** haemoglobin variants, idiopathic erythrocytosis, next‐generation sequencing, oxygen sensing pathway, variant prioritisation

## Abstract

Targeted next‐generation sequencing combined with a structured interpretative framework integrating gene–disease validity, population data, computational predictions, ACMG criteria and structural modelling enabled prioritisation of rare variants in idiopathic erythrocytosis, highlighting the genetic heterogeneity and biological complexity underlying this condition.

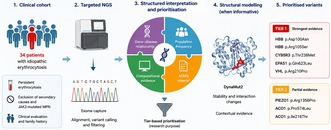

To the Editor,

Erythrocytosis is characterised by increased red blood cell mass arising from acquired or inherited causes. Secondary forms are commonly associated with hypoxia or neoplasms, whereas primary erythrocytosis most frequently reflects *JAK2*‐mutated polycythemia vera. However, a substantial proportion of patients lack an identifiable cause and are classified as having idiopathic erythrocytosis (IE)^1^. Germline variants affecting oxygen sensing and erythropoiesis are increasingly recognised, but interpretation of rare heterozygous variants remains challenging. Here, we applied targeted next‐generation sequencing (NGS) in IE using a structured prioritisation framework integrating gene–disease relationships, population data, computational predictions and structural modelling.^2^

Patients with persistent erythrocytosis were consecutively evaluated over a 3‐year period at our haematology clinic. Patients referred for evaluation of erythrocytosis had generally undergone preliminary investigations excluding secondary causes and Janus Kinase 2 (JAK2)‐mutated Myeloproliferative Neoplasms (MPN). Patients fulfilling criteria for IE were included. During the study period, 34 eligible patients were identified (Table 1). Erythrocytosis was defined as haemoglobin >16.5 g/dL in men or >16.0 g/dL in women, or haematocrit >0.49% and >0.48% respectively. Clinical evaluation included assessment of hypoxia‐related conditions, cardiopulmonary disease, medication history, arterial blood gases, overnight pulse oximetry, abdominal ultrasound and venous the oxygen tension when hemoglobin is 50 % saturated with oxygen (P50) estimation according to a validated model.^3^ The study was approved by the local ethics committee and conducted according to the Declaration of Helsinki. Genomic deoxyribonucleic acid (DNA) from peripheral blood was analysed by targeted NGS using exome capture technology (Illumina NextSeq 550). Variants were annotated using the eVai platform according to American College of Medical Genetics and Genomics (ACMG)/Association for Molecular Pathology (AMP) recommendations and interpreted using a predefined gene panel relevant to erythropoiesis and oxygen sensing (Table S1).^4^ Variants were prioritised using a tier‐based framework integrating gene–disease validity, population frequency, computational predictions and selected ACMG criteria. Structural modelling with DynaMut2 was used selectively as contextual rather than pathogenic evidence (see Supporting Information). Clinical characteristics of the cohort are summarised in Table 1. All detected variants are reported either in Table 2 (prioritised variants) or in Table S2 (non‐prioritised variants).

**TABLE 1:** Clinical characteristics of the entire erythrocytosis cohort.

Case	Age	Gender	High Ht since	Family history	Ht % range (L/L)	EPO (n.v.2.6‐18.5 U/L)	CVE
P#1	65	M	2022	Brother	0.52–0.54	13.2	DVT
P#2	68	M	2000	No	0.50–0.55	6.7	None
P#3	25	M	2016	No	0.50–0.53	9.4	None
P#4	30	M	2022	No	0.49–0.52	1.6	None
P#5	75	F	2006	Son	0.49–0.52	7.1	None
P#6	24	M	2015	No	0.47–0.53	10.7	None
P#7	73	F	2005	No	0.48–0.52	9.0	None
P#8	75	M	1990	Father and daughter	0.47–0.53	11.0	None
P#9	61	M	2002	No	0.51–0.55	7.4	None
P#10	63	F	2018	2 cousins	0.51–0.53	9.8	None
P#11	77	F	1977	Paternal grandmother, father, paternal uncle, daughter	0.49–0.54	11.7	None
P#12	60	M	1998	No	0.52–0.55	9.7	None
P#13	66	F	2014	No	0.50–0.53	3.2	None
P#14	50	M	2000	No	0.50–0.53	6.9	None
P#15	74	M	1995	No	0.51–0.55	10.3	None
P#16	61	M	2015	No	0.50–0.54	4.9	TIA
P#17	33	M	2010	No	0.51–0.56	5.8	None
P#18	75	M	2013	No	0.50–0.55	7.1	None
P#19	22	M	2019	No	0.52–0.54	4.3	None
P#20	80	M	2009	No	0.50–0.54	8.7	None
P#21	25	M	2022	No	0.52–0.57	5.4	None
P#22	39	F	2022	Mother	0.48–0.53	6	None
P#23	20	M	2024	No	0.49–0.52	4.9	None
P#24	34	M	2020	Mother	0.52–0.55	9	None
P#25	63	M	2009	No	0.52–0.56	7.2	None
P#26	61	F	2001	Father	0.49–0.51	6.5	None
P#27	74	F	2004	No	0.49–0.51	8.7	MI
P#28	53	M	1984	Father, maternal aunt	0.54–0.60	30	AF, DVT
P#29	26	M	2020	No	0.50–0.54	9.4	None
P#30	67	M	2003	No	0.47–0.56	9.4	None
P#31	83	M	2002	Brother	0.48–0.56	19.0	None
P#32	73	M	1984	No	0.47–0.56	17.0	None
P#33	61	M	2014	No	0.51–0.53	7.5	None
P#34	30	M	2023	No	0.50–0.54	3.8	None

Abbreviations: AF, atrial fibrillation; CVE, cardiovascular events; DVT, deep vein thrombosis; EPO, erythropoietin; MI, myocardial infarction; TIA, transient ischemic attack.

**TABLE 2:** Subgroup of patients with variants prioritised based on gene–disease relationship and tier‐based classification.

Variants	Patient #	Gene–disease relationship	GnomAD max AF	ACMG population criterion	Computational evidence	Additional ACMG criteria	Study tier (research prioritisation)
*HBB p*.Arg105Ser	P#27	Definitive	0.00041	PM2	PP3	PM1	Tier‐1
*HBB* p.Asp100Asn	P#28	Definitive	Absent	PM2	PP3	PM1	Tier‐1
*CYB5R3 p*.Thr238Met	P#29	Strong	0.000991	PM2	PP3	PM1	Tier‐1
*EPAS1 p*.Gln623Leu	P#2	Definitive	Absent	PM2	Conflicting	PM1	Tier‐1
*VHL* p.Arg210Pro	P#14	Definitive	Absent	PM2	Conflicting	PM1	Tier‐1
*PIEZO1 p*.Arg1356Pro	P#30	Strong	0.002542	—	Conflicting	PP2 (supporting)	Tier‐2
*ACO1 p*.Pro574Leu	P#31	Moderate	Absent	PM2	Conflicting	—	Tier‐2
*ACO1/IRP1* p.Ile216Thr	P#32	Moderate	Absent	PM2	Conflicting	PM1	Tier‐2

*Note*: Table 2 includes only patients harbouring variants prioritised according to predefined criteria. Variants identified in genes relevant to erythrocytosis that did not fulfil prioritisation criteria are reported in Table S2 but are not included here. Variant prioritisation was based on integration of gene–disease validity for erythrocytosis, population allele frequency, computational predictions, and—when informative—structural modelling. This framework was applied to distinguish variants with stronger biological plausibility from those considered less likely to contribute to the phenotype. ACMG/AMP criteria were used as a standardised descriptive framework but not as a surrogate for clinical pathogenicity classification. This prioritisation highlights the heterogeneity of genetic findings in IE.

Abbreviations: AF, atrial fibrillation; IE, idiopathic erythrocytosis.

Missense variants of the haemoglobin beta subunit (*HBB*) gene, encoding the β‐globin subunit of adult haemoglobin (HbA), may increase oxygen affinity, reducing oxygen delivery to tissues and leading to functional hypoxia. In this context, we identified the known pathogenic variant *HBB* Asp100Asn (Hb Kempsey),^5^ the only variant associated with elevated erythropoietin levels. Structural analysis showed a subtle reorganisation of the polar interaction network surrounding residue 100. In the wild‐type structure, Asp100 participates in a charge‐dependent interaction network involving Glu102 and Asn103 at the α1β2/α2β1 allosteric interface (Figure S1). Substitution with asparagine removes the negatively charged side chain, altering local polar interactions without major disruption of the overall network. DynaMut2 predicted a mild destabilising effect (ΔΔG = −0.29 kcal/mol). Despite subtle structural changes, this variant is well established as a high oxygen‐affinity haemoglobin. Similar β‐globin variants have been linked to familial erythrocytosis^6^.

A second *HBB* variant, Arg105Ser, was identified in another patient. Structural modelling indicated disruption of a multivalent interaction hub centred on residue 105. In the wild‐type configuration, Arg105 participates in an extensive network of polar and electrostatic interactions involving Glu102, Asn103, Asn109, Gly108 and Ala139 (Figure S2), acting as a central stabilising node linking neighbouring regions. Replacement with serine markedly reduces the extent and connectivity of this network, with loss of long‐range interactions and retention of only limited local contacts. DynaMut2 predicted a destabilising effect (ΔΔG = −0.44 kcal/mol), suggesting reduced local stability of the β‐globin chain. Although functional validation is lacking, these structural findings support a biologically plausible impact on haemoglobin function and therefore cautious consideration of a potential contribution to erythrocytosis.

We also identified a missense variant in *CYB5R3*, encoding cytochrome b5 reductase 3, an enzyme required for maintaining haemoglobin in its ferrous (Fe^2+^) state. The variant Thr238Met lies near the flavin‐binding region critical for enzymatic activity (Figure S3). Substitution with methionine alters the physicochemical nature of the residue, resulting in a shift from polar to predominantly hydrophobic interactions and a more dispersed pattern of contacts. Although overall structural stability is only mildly affected (ΔΔG = −0.3 kcal/mol), these changes may influence the local environment of the cofactor‐binding region. Heterozygous *CYB5R3* variants have been reported to impair methaemoglobin reduction in selected contexts and may contribute to erythrocytosis through functional hypoxia, supporting prioritisation of this variant as Tier 1.^7^

Two additional Tier 1 variants were identified in genes of the oxygen‐sensing pathway: Endothelial PAS Domain‐containing Protein 1 (*EPAS1)* Gln623Leu and Von Hippel Lindau (*VHL*) Arg210Pro. *EPAS1* encodes hypoxia‐inducible factor 2α, a key regulator of erythropoietin transcription, whereas *VHL* encodes the von Hippel–Lindau protein responsible for oxygen‐dependent degradation of hypoxia‐inducible factors. Variants in both genes are well‐established causes of congenital erythrocytosis. In our cohort, both variants met predefined prioritisation criteria based on gene–disease association and rarity; however, computational predictions were conflicting and structural analysis was not considered sufficiently informative.

Among Tier 2 variants, we identified the Piezo Type Mechanosensitive Ion Channel Component 1 (*PIEZO1)* missense variant Arg1356Pro in one patient. *PIEZO1* encodes a mechanosensitive ion channel involved in red cell hydration, and gain‐of‐function variants are associated with hereditary stomatocytosis and occasionally erythrocytosis. Structural modelling suggested disruption of the local interaction network centred on Arg1356, with marked reduction in connectivity within the 1352–1360 loop (Figure S4). Despite these local rearrangements, predicted global stability effects were minimal (ΔΔG = +0.03 kcal/mol). These findings support classification as a biologically plausible but less definitive candidate variant. Additional functional studies, including ektacytometry, may clarify the biological relevance of this PIEZO1 variant.

Two additional Tier 2 variants involved *ACO1* (iron regulatory protein 1), which coordinates iron metabolism with erythropoiesis and interacts with hypoxia signalling pathways. The Ile216Thr substitution lies near the [4Fe–4S] cluster‐binding region. In the wild‐type structure, Ile216 participates in an extended interaction network involving Asp199 and a broader interaction hub centred on Asp214, which connects the 211–218 loop to more distal residues (Figure S5). In the Thr216 model, the residue retains local contacts but no longer engages the Asp214‐centred network, resulting in a more localised interaction pattern. Consistently, DynaMut2 predicted a destabilising effect (ΔΔG = −2.44 kcal/mol), suggesting reduced structural cohesion in this region (Figure S5). A second variant, Pro574Leu, was associated with reorganisation of the local interaction pattern within this loop region (Figure S6). In the wild‐type structure, Pro574 contributes to a constrained loop conformation with focused interactions involving Gln583 and Gly576, whereas substitution with leucine results in a broader and less focal interaction network. DynaMut2 predicted a mild destabilising effect (ΔΔG = −0.34 kcal/mol).

While these findings suggest possible functional consequences, their clinical relevance remains uncertain. Venous P50 values were available for 29/34 patients and were within the normal range in all cases except in the patient harbouring the Hb Kempsey variant, who showed a reduced P50 consistent with increased oxygen affinity. Erythropoietin levels in our cohort were predominantly within the normal range, consistent with previous observations in IE. Four past thrombotic events were recorded, including one transient ischaemic attack, one myocardial infarction and two deep vein thromboses (Table 1). Non‐prioritised variants are summarised in Table S2.

IE remains a clinically and biologically heterogeneous condition defined largely by exclusion of secondary causes and clonal myeloproliferative neoplasms. NGS has expanded variant detection but shifted the main challenge towards interpretation, with frequent identification of rare variants of uncertain significance. Recent NGS‐based studies have similarly highlighted the prevalence of rare variants and the challenges of interpretation in erythrocytosis.^8^ Established mechanisms include altered haemoglobin oxygen affinity, impaired redox homeostasis and dysregulated oxygen sensing through *VHL* and *EPAS1* variants.^1^, ^9^, ^10^, ^11^, ^12^ Together, these mechanisms may impair oxygen delivery or sensing, leading to compensatory erythropoiesis even in the absence of overt erythropoietin elevation. Accordingly, erythropoietin levels in our cohort were predominantly within the normal range, consistent with inappropriately normal values described in IE. Beyond these mechanisms, emerging evidence supports roles for genes involved in iron metabolism and red cell membrane or ion transport. Proteins such as *ACO1*, integrating iron homeostasis with hypoxia signalling and *PIEZO1*, regulating red cell hydration, illustrate how heterogeneous genetic alterations may converge on common biological processes influencing erythropoiesis.^12^, ^13^, ^14^ In this context, reliance solely on ACMG/AMP classification criteria may be insufficient. While essential for monogenic disorders, their strict application to IE—where oligogenic contributions and incomplete penetrance are plausible—may lead to overclassification.^15^ Our tier‐based prioritisation strategy was therefore designed as a research‐oriented framework integrating gene–disease relationships, population data, computational evidence and selective structural modelling, used cautiously as contextual rather than definitive evidence.^16^ This approach may be relevant in clinical settings where NGS identifies multiple rare variants, helping to prioritise those most likely to warrant functional validation or clinical follow‐up. Overall, IE likely reflects heterogeneous genetic perturbations affecting convergent biological pathways. Structured prioritisation approaches may improve interpretative consistency and facilitate identification of candidates for functional validation.

## AUTHOR CONTRIBUTIONS

FV conceived and supervised the study, coordinated patient recruitment and oversaw the clinical and genetic workflow. FV and GC designed the genetic analysis strategy and supervised variant interpretation and prioritisation. AG, AM, JC, GG, MP and SC performed sample processing, next‐generation sequencing procedures and laboratory‐based experimental work. GC, AG and AM contributed to the supervision of the genetic pipeline and tertiary analysis. AC, AV, CG, VC, FC, FP, GB, IG, LT, MC, ND, SZ and WA contributed to clinical assessment, patient management and phenotypic data collection. FV drafted the manuscript. All authors contributed to data interpretation, critically revised the manuscript for important intellectual content, and approved the final version of the manuscript.

## FUNDING INFORMATION

The authors declare that no funds, grants, or other support were received during the preparation of this manuscript.

## CONFLICT OF INTEREST STATEMENT

The authors have no relevant financial or non‐financial interests to disclose.

## ETHICS STATEMENT

This study was performed in line with the principles of the Declaration of Helsinki. Approval was granted by the local Ethics Committee (ref. AOP3957).

## PATIENT CONSENT STATEMENT

Informed consent was obtained from all individual participants included in the study.

## Supporting information


Figure S1.
Figure S2.

Figure S3.
Figure S4.

Figure S5.
Figure S6.

Table S1.
Table S2.

## Data Availability

The data that support the findings of this study are available on request from the corresponding author. The data are not publicly available due to privacy or ethical restrictions.
