# Supplementary material for: From variant detection to interpretation in idiopathic erythrocytosis: A structured approach applied to a clinical cohort
Source: Br J Haematol. 2026 Jun 12;209(2):837–41. doi: 10.1111/bjh.70597 (PMC13461990; doi:10.1111/bjh.70597)
Supplement: Supplementary file 1 [file BJH-209-837-s004.docx]

**Supplemental information**

**Next-generation sequencing**

DNA genomic was extracted from each participant using QIAamp DNA Mini kit (Qiagen,Germany) from peripheral venous blood. Whole exonic regions were captured by xGen Exome Hyb Panel v2. (IDT, USA) which consists of 415,115 individually synthesized, 5’ biotinylated, oligonucleotide probes. The libraries for targeted capture were prepared using the xGen™ NGS DNA Library Preparation (IDT, USA). Briefly, 100 ng of DNA was quantified by Qubit DNA HS assay (Life Technologies, Milan, Italy). Genomic DNA was enzymatically fragmented and then ligated with stubby P5 and P7 adapters. AMPure XP beads (Beckman Coulter, Brea, CA, USA) were used to purify the libraries, which were quantified by LabChip GX Touch Nucleic Acid Analyzer (PerkinElmer, Boston, MA, USA). A multiplexing pool of 500 ng of each library from 8 samples was used for hybrid capture with the biotinylated DNA oligonucleotide probes. After hybridization, Streptavidin Dynabeads were used to capture biotinylated DNA. Purified DNA was amplified using the Bio-Rad CX200 thermal cycler (Bio-Rad Laboratories, Hercules, USA). The library pool was further purified using AMPure XP beads (Beckman Coulter, Brea, USA) and quantified by Qubit DNA HS assay. Amplicon length average size was 254. Finally, libraries were sequenced on Next Seq 550 with High Output flow cell (300 cycles) at a depth of 50 × , coverage of 99.99%  (Illumina, USA).

**Primary bioinformatic analysis**

A file detailing the sequence of the hybridizing oligonucleotides, and the coordinates of designed amplicons, downloaded from the manufacturer (IDT, Tema Ricerca, Milan, Italy) was uploaded into the CLC genomics Workbench (vers. 25.0.1, Qiagen, Germany). The sequencing reads were aligned to the human reference sequence GRCh38/hg38, and the workflow then removed ligation artifacts from the read mapping. Next, the Indels and Structural Variants detection step generated a guidance track used for improving the mapping with the Local Realignment tool. Variant allele frequency (VAF) was then detected using the Fixed Ploidy Variant Detection tool for germline applications (> 30%). Finally, a series of filtering steps was applied to remove variants that likely entailed artifacts. The final output of the workflow produces a list of filtered variants and BAM files which are visualized in IGV (Integrative Genomics Viewer).

**Variant annotation and interpretative framework**

Variants were annotated and initially interpreted using the eVai (Expert Variant Interpreter; enGenome, Italy) platform, integrating population frequency, predicted functional impact, and available clinical information according to ACMG/AMP recommendations. ACMG classification was used as a standardized interpretative language but not as a surrogate for definitive clinical diagnosis.

Analysis focused on a predefined panel of genes involved in erythropoiesis, oxygen sensing, hemoglobin structure and function, red cell metabolism, and related pathways, as reported in Supplemental Table 1.

**Gene–disease relationship assessment**

Gene–disease validity for erythrocytosis was assessed prior to variant-level interpretation. Genes were classified as having definitive, strong, moderate, or limited evidence for a causal role in erythrocytosis based on ClinGen criteria where available, or on published human genetic evidence and biological plausibility. Only variants identified in genes with definitive, strong, or moderate evidence were considered eligible for tier-based prioritisation.

**Population frequency and computational evidence**

Population allele frequencies were assessed using gnomAD, considering the maximum allele frequency across all ancestries. Population-based ACMG criteria were applied as follows: PM2 for variants absent from gnomAD or with maximum allele frequency ≤0.1%; BS1 for frequencies ≥0.5%; and BA1 for frequencies ≥5%. Variants with intermediate frequencies were not assigned population-based criteria.

Computational predictions were used as supporting evidence only. Multiple in silico tools were evaluated (REVEL, CADD, PolyPhen-2, SIFT, PaPI). PP3 was assigned when predictions consistently supported a deleterious effect, while BP4 was assigned for consistently benign predictions. Computational evidence alone was not considered sufficient to infer pathogenicity.

**Tier-based variant prioritisation**

Variants were stratified into study-specific tiers for research prioritisation purposes only, distinct from ACMG clinical classification.

Tier 1 variants were defined as variants identified in genes with definitive or strong evidence for erythrocytosis, fulfilling PM2, PP3, and at least one additional ACMG criterion (e.g. PM1 or PS1). Tier 2 variants included variants with partial supporting evidence that did not meet all Tier 1 criteria. Variants not fulfilling predefined prioritisation criteria or identified in genes with limited gene–disease validity were not prioritised for downstream analyses.

In addition to population frequency and computational evidence, selected ACMG/AMP criteria were considered solely as a standardized descriptive framework to document biological and functional features supporting tier-based variant prioritisation. These criteria were applied individually, without combination, and were not used to derive clinical ACMG variant classifications or to infer pathogenicity.

**Structural modelling and Sanger validation**

Structural modelling and stability predictions were performed using DynaMut2, when structural analysis was expected to provide additional interpretative value. High-confidence protein structures were obtained from the Protein Data Bank or the AlphaFold Protein Structure Database. Changes in protein stability (ΔΔG) and non-covalent interactions were assessed by comparison of wild-type and mutant models. Structural modelling was applied selectively when expected to provide mechanistic insight, including in variants with conflicting predictions.

**Supplementary Table 1**: Gene Selected for Erythrocytosis phenotype frow WES Sequencing

| **Gene** | **Name** | **Chromosome** | **Pathway** |
| --- | --- | --- | --- |
| *EGLN1* | Egl-9 Family Hypoxia Inducible Factor 1 | 1 |  |
| *EGLN2* | Egl-9 Family Hypoxia Inducible Factor 2 | 19 | Hypoxia-sensing Pathway |
| *EGLN3* | Egl-9 Family Hypoxia Inducible Factor 3 | 14 |  |
|  |  |  |  |
| *HIF-1α* | Hypoxia Inducible Factor 1 Subunit Alpha | 14 |  |
| *HIF-2α (EPAS1)* | Hypoxia Inducible Factor 1 Subunit Alpha | 2 |  |
| *HIF-3α* | Hypoxia Inducible Factor 1 Subunit Alpha | 19 | Hypoxia Pathway |
| *HIF1AN* | Hypoxia Inducible Factor 1 Inhibitor | 10 |  |
| *VHL* | Von Hippel-Lindau Tumor Suppressor | 3 |  |
| *BHLHE41* | Basic Helix-Loop_Helix E41 | 12 |  |
|  |  |  |  |
| *EPO* | Erythropoietin | 7 |  |
| *EPOR* | Erythropoietin Receptor | 19 |  |
| *JAK2* | Janus Kinase 2 | 9 |  |
| *GH* | Growth Hormone 1 | 17 | Erythropoietin Pathway |
| *GH1* | Growth Hormone 2 | 17 |  |
| *CSH1* | Chorionic Somatomammotropin Hormone 1 | 17 |  |
| *CSH2* | Chorionic Somatomammotropin Hormone 1 | 17 |  |
|  |  |  |  |
| *BPGM* | Bisphosphoglycerate Mutase | 7 |  |
| *HBA1* | Hemoglobin Subunit Alpha 1 | 16 | Oxygen affinity of hemoglobin |
| *HBA2* | Hemoglobin Subunit Alpha 1 | 16 |  |
| *HBB* | Hemoglobin Subunit Beta | 11 |  |
|  |  |  |  |
| *PFKM* | Phosphofructokinase | 12 |  |
| *PKLR* | Pyruvate Kinase | 1 | Glycolysis Pathway |
| *HK1* | Hexokinase 1 | 10 |  |
|  |  |  |  |
| *ANK1* | Ankyrin 1 | 8 |  |
| *ANKRD26* | Ankyrin Repeat Domain Containing 26 | 10 |  |
| *SPTA1* | Spectrin alpha 1 | 1 |  |
| *SPTB* | Spectrin beta1 | 14 |  |
| *SLC4A1* | Solute Carrier Family 4 Member 1 | 17 |  |
| *PIEZO1* | Piezo Type Mechanosensitive 1 | 16 | Erythrocyte Cytoskeleton |
| *EPB41* | Erythrocyte Membrane Protein Band 4.1 | 1 |  |
| *EPB42* | Erythrocyte Membrane Protein Band 4.2 | 15 |  |
| *KCNN4* | Potassium Calcium-Activated 4 | 19 |  |
| *RHAG* | Rh blood group, glycoprotein A | 6 |  |
| *ACKR1* | Atypical Chemokine Receptor 1 | 1 |  |
|  |  |  |  |
| *ACO1* | Aconitase1 | 9 |  |
| *HFE* | Homeostatic Iron Regulator | 6 |  |
| *CYB5A* | Cytochrome B5 Type A | 18 |  |
| *CYB5R3* | Cytochrome B5 Reductase 3 | 22 |  |
| *BMP2* | Bone Morphogenetic Protein 2 | 20 |  |
| *FTH1* | Ferritin Heavy Chain 1 | 11 | Iron Metabolism |
| *HAMP* | Hepcidin | 19 |  |
| *HJV* | Hemojuvelin | 1 |  |
| *SLC40A1* | Solute Carrier Family 40 Member 1 | 2 |  |
| *TFR2* | Transferrin Receptor 2 | 7 |  |
|  |  |  |  |
| *GFI1B* | Growth Factor 1B Transcriptional Repressor | 9 |  |
| *SH2B3* | SH2B Adaptor Protein 3 | 12 | Hematopoiesis |
| *SOCS2* | Suppressor Of Cytokine Signaling 2 | 12 |  |
| *SOCS3* | Suppressor Of Cytokine Signaling 3 | 17 |  |

**Supplementary Table 2.** Non-prioritised variants identified by next-generation sequencing, reported for each patient in the same order as Table 1. Variants already included in Table 2 (Tier-1 and Tier-2) are not shown. Remaining variants are grouped according to whether the affected gene has an established or biologically plausible association with erythrocytosis.

| **Case** | **Variants in genes with established or plausible association with erythrocytosis (not prioritised)** | **Variants in genes without established association with erythrocytosis** |
| --- | --- | --- |
| P#1 | *PIEZO1* Glu2392Lys; *PIEZO1* Arg2336Gln | *ACKR1* Arg89Cys |
| P#2 | *EPAS1* Gln623Leu *EGLN1* (PHD2) Cys127Ser | *HFE* intronic variant (rs2071303) |
| P#3 | — | *HIF3A* Ala401Val *SLC4A1* Arg387Gln |
| P#4 | — | *EPO* Ala151Ala |
| P#5 | *PFKM* stop coding in Glu5Ter | *HIF1A* Ile607Met *HFE* His63Asp *HFE* intronic variant (rs2071303) |
| P#6 | — | *SPTA1* Arg1414Cys *HFE* Ser65Cys |
| P#7 | *EPAS1* Phe374Tyr | *SPTA1* Arg137His *HFE* Ser65Cys |
| P#8 | — | *SH2B3* Trp262Arg |
| P#9 | — | *HFE* His63Asp |
| P#10 | — | *SPTA1* His415Tyr *ANK1* Ala1553Val |
| P#11 | *PIEZO1* Gln1468_Ala1469insGluGln | *SPTA1* His415Tyr |
| P#12 | — | *HFE* His63Asp |
| P#13 | *VHL* Pro81Ser | — |
| P#14 | — | — |
| P#15 | — | *HFE* His63Asp |
| P#16 | — | — |
| P#17 | — | *SH2B3* Trp262Arg*; ANKRD26* Val1305Ile |
| P#18 | *PIEZO1* Lys2158Arg | *HFE* His63Asp |
| P#19 | — | *SPTA1* Gln427Pro |
| P#20 | — | *HFE* His63Asp |
| P#21 | *PIEZO1* Leu415Val | *EPO* Ala151Ala |
| P#22 | *HK1* Arg396Cys | — |
| P#23 | *EPAS1* Phe374Tyr | *SPTB* Ala1673Thr |
| P#24 | — | *TFR2* Asp726Asn; *ANKRD26* Glu1562Lys |
| P#25 | — | *HFE* His63Asp |
| P#26 | — | *HFE* Cys282Tyr |
| P#27 | — | — |
| P#28 | — | — |
| P#29 | — | — |
| P#30 | — | — |
| P#31 | — | — |
| P#32 | — | — |

**Legends to figures**

**Figure 1.** Structural comparison of wild-type and mutant β-globin at residue 100.

The local interaction network surrounding residue 100 is shown for the wild-type Asp100 (left panel) and the mutant Asn100 (right panel). In the wild-type structure, Asp100 participates in a charge-dependent interaction network involving Glu102 (magenta dashed line) and additional polar contacts (orange dashed lines) within the 100–103 cluster. Substitution with asparagine removes the negatively charged side chain, resulting in a subtle reorganisation of local polar interactions without major disruption of the overall network. DynaMut2 predicts a mild destabilising effect (ΔΔG = −0.29 kcal/mol).

**Figure 2.** Comparison of the local interaction network surrounding residue 105 in the wild-type β-globin (Arg105, left panel) and the Arg105Ser mutant (right panel). In the wild-type structure, Arg105 participates in multiple stabilizing polar and electrostatic interactions (red dashed lines) with Glu102, Asn103, and Asn109, and additional contacts (orange and green dashed lines) with Gly108 and Ala139, forming a multivalent interaction hub. In the Ser105 mutant, the extent and connectivity of this network are markedly reduced, with loss of long-range interactions and retention of only limited local contacts. DynaMut2 predicts a destabilizing effect of the mutation (ΔΔG = −0.44 kcal/mol), consistent with disruption of the local interaction network.

**Figure 3.** Structural comparison of the wild-type (Thr238, left panel) and mutant (Met238, right panel) *CYB5R3* structures. In the wild-type configuration, Thr238 participates in a network of polar interactions (dashed lines) with Gly249, Gln250, Gly251, Ala209, and Asn210, contributing to the local interaction environment within the FAD-binding region. In the mutant structure, substitution with methionine reduces hydrogen-bonding capacity and is associated with increased hydrophobic contacts with nearby residues, including Leu207, Ala209, Val253, and Thr257. This results in a reorganisation of the local interaction network, with a shift from polar to more hydrophobic contacts. The overall fold appears preserved, while local rearrangements may influence the microenvironment of the binding region.

**Figure 4.** Structural comparison of the wild-type (Arg1356, right panel) and mutant (Pro1356, left panel) *PIEZO1* residue highlights a pronounced reorganization of the local interaction network. In the wild-type structure, Arg1356 engages in multiple stabilizing polar interactions (dashed lines) with Gln1353, Met1354, Ala1359, Lys1360, and Arg1358, forming a multivalent interaction hub that integrates the 1352–1360 loop with a more distal interaction cluster involving Asp2483. Replacement of arginine with proline reduces hydrogen-bonding capacity and introduces conformational rigidity, leading to a marked reduction in the connectivity of the interaction network and reduced engagement of both local and more distal contacts. Although global stability is predicted to be largely preserved, these local changes support a potentially relevant structural impact.

**Figure 5.**  Structural comparison of the wild-type (Ile216, left panel) and mutant (Thr216, right panel) *ACO1* residues reveals a reorganization of the local interaction network. In the wild-type structure, Ile216 contributes to local packing interactions involving Asp199 and participates in a broader interaction network centred on Asp214, which connects the 211–218 loop to more distal residues such as Ala186 and Val188. This extended network integrates the loop into the surrounding structural environment. In the Thr216 mutant, the residue retains a limited set of local interactions but no longer engages the Asp214-associated network, resulting in a more localised and less integrated interaction pattern. This is consistent with the destabilizing effect predicted by DynaMut2 (ΔΔG = −2.44 kcal/mol).

**Figure 6.** Local interaction network surrounding residue 574 in the wild-type (Pro574, left panel) and mutant (Leu574, right panel) *ACO1* structures. In the wild-type configuration, Pro574 contributes to a locally constrained loop conformation, promoting a compact interaction pattern involving Gln583 and Gly576. In the Leu574 mutant, replacement of proline alters local conformational constraints, resulting in a broader and more dispersed set of interactions with reduced focal connectivity. Overall, the interaction network appears less localised, consistent with the modest destabilizing effect predicted by DynaMut2 (ΔΔG = −0.34 kcal/mol)
